# Single-crystal two-dimensional material epitaxy on tailored non-single-crystal substrates

**DOI:** 10.1038/s41467-022-29451-w

**Published:** 2022-04-01

**Authors:** Xin Li, Guilin Wu, Leining Zhang, Deping Huang, Yunqing Li, Ruiqi Zhang, Meng Li, Lin Zhu, Jing Guo, Tianlin Huang, Jun Shen, Xingzhan Wei, Ka Man Yu, Jichen Dong, Michael S. Altman, Rodney S. Ruoff, Yinwu Duan, Jie Yu, Zhujun Wang, Xiaoxu Huang, Feng Ding, Haofei Shi, Wenxin Tang

**Affiliations:** 1 Chongqing Key Laboratory of Multi-Scale Manufacturing Technology, Chongqing Institute of Green and Intelligent Technology, Chinese Academy of Sciences, Chongqing, 400714 P.R. China; 2 University of Chinese Academy of Sciences, No.19(A) Yuquan Road, Shijingshan District Beijing, 100049 P.R. China; 3 International Joint Laboratory for Light Alloys (MOE), College of Materials Science and Engineering, Chongqing University, Chongqing, 400044 P.R. China; 4 Beijing Advanced Innovation Center for Materials Genome Engineering, University of Science and Technology Beijing, Beijing, 100083 P.R. China; 5 Center for Multidimensional Carbon Materials (CMCM), Institute for Basic Science (IBS), Ulsan, 44919 Republic of Korea; 6 Department of Materials Science and Engineering, Ulsan National Institute of Science and Technology (UNIST), Ulsan, 44919 Republic of Korea; 7 Electron Microscope Center, Chongqing University, Chongqing, 400044 PR China; 8 Department of Physics, The Hong Kong University of Science and Technology (HKUST), Clear Water Bay, Kowloon, Hong Kong, PR China; 9 School of Materials Science and Engineering, Ulsan National Institute of Science and Technology (UNIST), Ulsan, 44919 Republic of Korea; 10 School of Energy and Chemical Engineering, Ulsan National Institute of Science and Technology (UNIST), Ulsan, 44919 Republic of Korea; 11 Chongqing Key Laboratory of Graphene Film Manufacturing, Chongqing, 401329 P.R. China; 12 Shanghai Tech University, 93 Middle Huaxia Road, Pudong, Shanghai, 201210 P.R. China; 13 Shenyang National Laboratory for Materials Science, Chongqing University, Chongqing, 400044 P.R. China

**Keywords:** Two-dimensional materials, Metals and alloys, Electronic properties and devices

## Abstract

The use of single-crystal substrates as templates for the epitaxial growth of single-crystal overlayers has been a primary principle of materials epitaxy for more than 70 years. Here we report our finding that, though counterintuitive, single-crystal 2D materials can be epitaxially grown on twinned crystals. By establishing a geometric principle to describe 2D materials alignment on high-index surfaces, we show that 2D material islands grown on the two sides of a twin boundary can be well aligned. To validate this prediction, wafer-scale Cu foils with abundant twin boundaries were synthesized, and on the surfaces of these polycrystalline Cu foils, we have successfully grown wafer-scale single-crystal graphene and hexagonal boron nitride films. In addition, to greatly increasing the availability of large area high-quality 2D single crystals, our discovery also extends the fundamental understanding of materials epitaxy.

## Abstract (web summary)

The heteroepitaxial growth of crystalline 2D materials is usually based on single-crystal substrates. Here, the authors demonstrate that single-crystal graphene and hexagonal boron nitride films can be successfully grown on wafer-scale copper foils with tailored twin boundaries.

## Introduction

Wafer-scale single-crystal two-dimensional (2D) materials are highly desirable for the next generation of 2D material-based integrated electronics, optoelectronics and spintronics^1–5^. By judiciously choosing single crystal substrates with the appropriate surface texture, epitaxial growth of large area single-crystal graphene, hexagonal boron nitride (hBN), and other two-dimensional (2D) materials have recently been realized^6–16^. Despite significant progress in the synthesis of single-crystal substrates, such as Cu foils with different surface indices^17–19^, the preparation of single-crystal substrates is still generally expensive. Thermodynamically stable twin boundaries are common in face centered cubic (FCC) metals and are easily created during annealing treatments^20–22^. The presence of highly stable twin boundaries in Cu foils makes synthesizing high quality Cu single crystals challenging, and if possible, using polycrystalline substrates for 2D materials epitaxy would be highly beneficial.

Here we present a theoretical prediction and experimental demonstration of epitaxial growth of single-crystal 2D materials on tailored non-single-crystal substrates. We have successfully annealed polycrystalline Cu foils into various types of inch sized twinned Cu foils. Following the theoretical prediction, inch-sized 2D materials (including graphene and hBN) single crystals on selected twinned Cu foils have been synthesized. This study opens a rapid, scalable and robust route for single crystal 2D materials growth on tailored polycrystalline substrates.

## Results

### Theoretical frame for alignment of 2D materials on twinned Cu foils

**Fig. 1:**
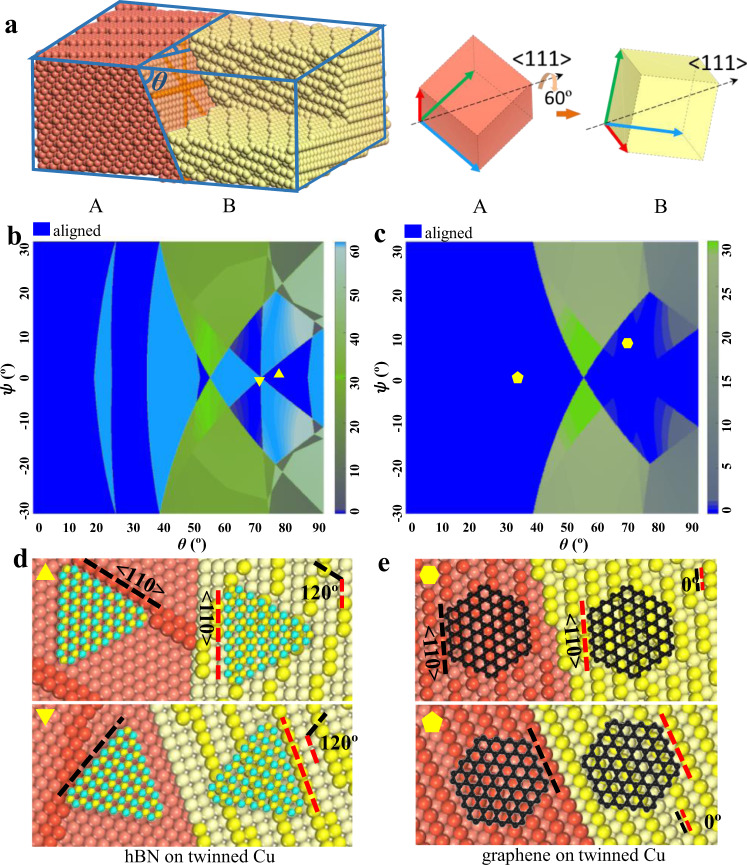
2D materials alignment on twinned Cu substrates. **a** Atomic configuration of a 60° <1 1 1> twin in a Cu foil and the definition of the two degrees of freedom, (θ, *ψ*), of the twin structure. Where θ is the angle between the Cu foil surface and the twin boundary and *ψ* is the in-plan rotation angle of the twin plane. The crystal axials of the two crystal lattices on both sides of the twin boundary (A and B) and their <1 1 1> co-axis is also shown. **b** Theoretical map of the misalignment angles between 3-fold symmetric 2D materials grown on the two sides of FCC twins as a function of (θ, *ψ*), which define the facet indices of the twinned surface. Colour bar shows the misaligned angles between 2D materials grown on twinned crystal surfaces and blue indicates perfect alignments. **c** Theoretical map of the misalignment angles between 6-fold symmetric 2D materials, such as graphene, grown on both sides of FCC twins as a function of (θ, *ψ*). Colour bar shows the misaligned angles of 2D islands grown on the twinned crystals and blue denotes perfect alignment. We can clearly see that the unidirectional alignment of 2D materials with either 3-fold or 6-fold symmetry can be realized on a large number of twinned FCC crystals. So, in principle, this should allow us to use a variety of twinned substrates to grow 2D single crystals. **d** Schematic diagram of hBN epitaxy on twinned Cu substrates, Cu atoms of step edges are highlighted. The alignment directions of hBN islands are denoted by dashed lines. **e** Schematic diagram of graphene epitaxy on twinned Cu substrates, Cu atoms of step edges are highlighted. The alignment directions of graphene islands are denoted by dashed lines.

The epitaxial growth of a 2D single crystal on a polycrystalline substrate requires that all 2D islands grown on two sides of a grain boundary are perfectly aligned, which is obviously impossible for an arbitrary grain boundary. Let’s consider the alignment of 2D material islands on the two sides of a 60° <111> twin boundary. The schematic diagram of a 60° <111> twin of a Cu foil with FCC structure is shown in Fig. 1a with more details in Supplementary Fig. 1. Here, the angle between the twin plane and the foil surface, θ, and the in-plan rotation angle of the twin plane, *ψ*, are the two independent variables that fully describe the twinned structure. In the Supplementary Notes 1–2 and Supplementary Figs. 2 and 3, we demonstrate that (θ, *ψ*) are equivalent to Euler angles or surface indices for determining the orientation of a twinned crystal and their transformation. Such a twin boundary carries extremely low formation energy and can rarely be avoided during annealing of FCC metals^20–22^. The position of some typical Cu twins containing at least one low-index surface plane in the (θ, *ψ*) map are presented in Supplementary Fig. 2c.

To explore the alignment of 2D islands on twinned substrates, it is necessary to know the principle that governs the alignment of 2D materials on arbitrary surfaces. Supplementary Fig. 4a shows two arbitrary (h k l) surfaces of an FCC crystal containing steps. Such steps are known to be active nucleation sites for 2D materials^23–27^. Previous studies reported that the alignment of 2D material (such as graphene, hBN) islands on a single-crystal Cu (1 1 1) surface is determined by the alignment of a zigzag edge of the 2D island with respect to a Cu <110> step edge^28,29^. However, an arbitrary FCC Cu surface may have many other types of step edges (<112>, <123>, etc.). Taking all these possible step edges into consideration makes it complicated to a priori determine the most favorable alignment of 2D material islands.

To understand the mechanism of alignment of 2D material islands on an arbitrary Cu surface, we investigated the stability of the interfaces of various 2D material edges with different metal step edges^30,31^. Taking graphene as an example, the interfacial formation energies of various graphene edge with respect to different metal step edge are calculated, see Supplementary Note 3 and Supplementary Fig. 4b. The deep valley observed along the diagonal of the map corresponds to an epitaxial relationship between graphene and the Cu surface. Based on this result, we deduced a simple geometric principle of graphene alignment on Cu surfaces. Where the most stable interfaces correspond to a graphene zigzag edge attaching to the longest (most predominant) <110> step edge segment of the Cu surface. More details are shown in the Supplementary Notes 4–5.

Here we selected the growth of two representative 2D materials, 3-fold symmetric hBN and 6-fold symmetric graphene, on twinned FCC Cu substrates as examples to demonstrate well-aligned 2D islands on both sides of a twin boundary. Including all possible twinned Cu foils, we calculated maps of misalignment angles between 2D material islands on the two sides of twin boundaries and the results are shown in Fig. 1b, c (see Supplementary Tables 1 and 2 for details). Figure 1d, e shows the predicted alignments of hBN and graphene islands on the two twinned Cu foil surfaces, where perfect alignment of the 2D islands on the twinned Cu surfaces is clearly seen. Our theoretical calculations predict that twinned surfaces that enable template-based epitaxial growth of hBN and graphene occupy significant portions of the maps, as shown by the blue regions in Fig. 1b, c (40.2% and 56.7% for hBN and graphene). This result shows that a majority of twinned Cu surfaces can be used as substrates for the uniaxial alignment of 2D material islands. Besides Cu substrates, the alignment maps (shown in Fig. 1b, c) are also applicable for graphene and hBN grown on other twin FCC metal substrates if the epitaxial relationship of a zigzag direction of a 2D material aligning along a high symmetric <110> direction of a substrate stands^32,33^. For example, Au substrates are promising for the epitaxial growth of transition metal dichalcogenides (TMDCs)^34,35^, sharing the same epitaxial relationship as that of hBN on Cu surface. Due to the same 3-fold symmetry of TMDCs and hBN crystal lattices, the alignment TMDCs on a twinned Au substrate is the same as that of hBN on a twinned Cu substrate of same type and, thus, the synthesis of single-crystal TMDCs on twinned Au surfaces is possible.

### Preparation of polycrystalline Cu foils

**Fig. 2:**
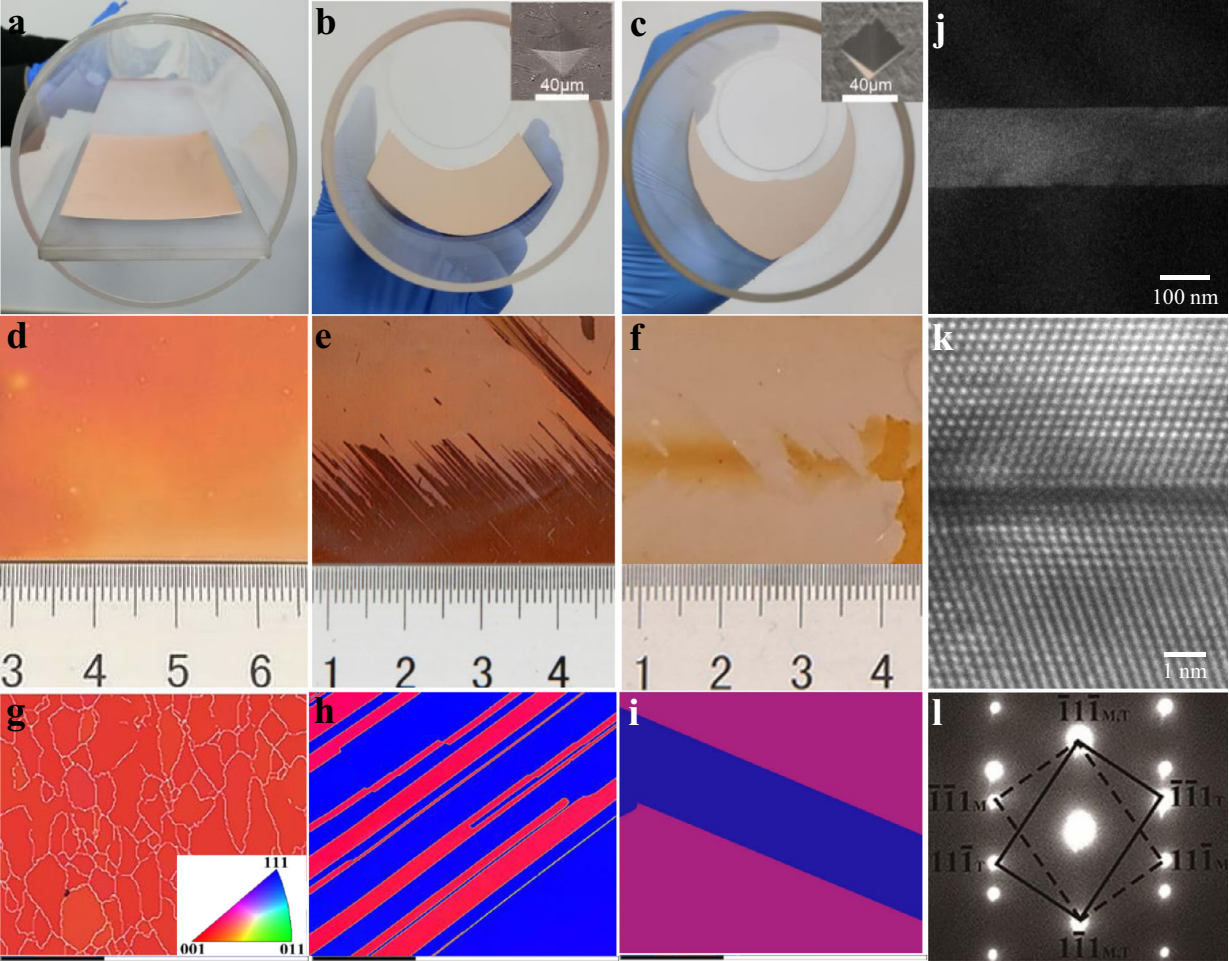
Cu foils tailoring configurations. **a**–**c** Photographs of the annealing experiment setup. **d**–**f** Optical images of annealed Cu foil in (**a**–**c**) after oxidation in air. **g**–**i** EBSD characterization corresponding to (**d**–**f**). The scale bar is 500 μm. **a** Annealing a flat Cu foil. EBSD mapping indicates that the cold rolled foil has been completely recrystallized, forming a polycrystal with relatively fine grain sizes (average grain size ~300 µm) and a strong Cube texture {001}<100>. A small fraction of twinned grains (blue patch) is also seen. **b** Annealing a bent Cu foil with a microhardness indentation in the center. Introducing an indent is an efficient way to stimulate controlled nucleation and growth during recrystallization; this technique has been used in previous recrystallization and growth studies. EBSD measurements show inch-scale (1 1 6)/(1 1 1) twinned structures. **c** Annealing a 45° rotated bent Cu foil with a microhardness indentation in the center. EBSD measurements show inch-scale (6 5 5)/(10 2 1) twinned structures. **j**–**l** TEM analysis of the obtained twinned Cu structure. Low magnification TEM, high resolution TEM and SAED images of a typical Cu twin crystal are displayed. The nature of coherent twins is confirmed. Insets in (**b**) and (**c**) are scanning electron microscopy (SEM) images of the indentation.

Experimentally, a variety of twin structures can be obtained by subjecting raw Cu foils to deformation and annealing treatments. Figure 2a, d, g show the results of annealing a flat Cu foil, where electron backscatter diffraction (EBSD) mapping shows that the cold rolled foil forms a strong cube textured {001}<100> polycrystal^22,36,37^ associated with a small twinned grain. If an indented Cu foil is bent along the transvers direction (TD)^38–41^, only one kind of twin structure is formed in the whole foil, as exhibited in Fig. 2b, e, h. Over the entire area of the foil, only two different optical intensities are seen, which arise from the different oxidization rates of the two different crystal orientations. EBSD mapping indicates that the dark and light areas in the optical image are approximately (1 1 6) and (1 1 1) surfaces, respectively. Except for the twin boundaries between these two areas, no other grain boundaries were detected in the foil. The pole and inverse pole figures in Supplementary Fig. 5 also confirm that no other orientations were present in the foil, implying that the inch-size Cu foil contained only (1 1 6)/(1 1 1) twin structures. To verify the role of the Cu foil surface structure in the annealing process, we selected another commercial polycrystalline copper foil for tailoring and obtained very similar results. This suggests that the key factors of twin boundary annealing are the bending and the indentation treatments, while the initial structure of the Cu foil is less important.

In a similar manner, by changing the experimental parameters (such as radius of curvature, indentation type and depth, and rotation angle), a variety of twin structures can be obtained. For example, if an indented Cu foil was bent along a direction 45° to the TD direction, the whole foil showed another kind of twin structure (6 5 5)/(10 2 1), as shown in Fig. 2c, f, i. The nature of coherent twins is confirmed by the transmission electron microscopy (TEM) in Fig. 2j–l.

### 2D materials growth and characterization

**Fig. 3:**
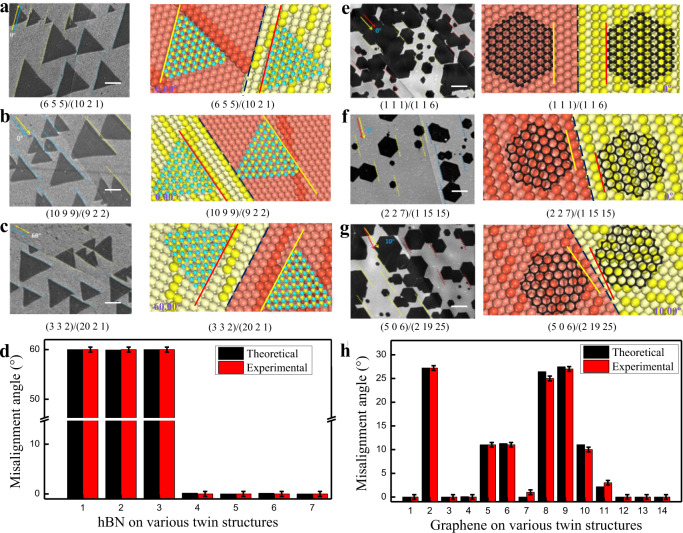
Alignment of hBN and graphene islands on different twinned Cu surfaces. SEM images of typical 2D material alignment experimentally observed on twinned Cu surface (left panels) compared with theoretical prediction (right panels). **a**–**c** are typical hBN islands grown on twinned Cu surfaces, in which all hBN islands shown in (**a**) and (**b**) are parallelly aligned while those shown in (**c**) show antiparallel alignment. **d** the theoretical prediction and experimental statistics of the misalignment angle of hBN grown on different substrates (The error bar is the standard deviation of raw data.). **e–g**, are typical graphene islands grown on twinned Cu surfaces, in which (**e**) and (**f**) show parallelly aligned graphene islands while islands in (**g**) are misaligned on both sides of the twin boundary. The angle between the 2D island edge and the twin boundary is marked in the corresponding theoretical diagram. Surface indices of the twin crystals are shown in each figure. The scale bars in all graphs are 10 μm. **h** the theoretical prediction and experimental statistics of the misalignment angles of graphene grown on different substrates (The error bar is the standard deviation of raw data.).

Figure 3 shows experimentally observed and theoretically predicted hBN and graphene islands grown on different twinned Cu surfaces. Figure 3a–b and e–f show perfectly aligned hBN and graphene on four different twinned Cu surfaces. Two more cases where we observed the formation of misaligned hBN and graphene are presented in Fig. 3c, g, and more results with various twin boundaries are shown in Supplementary Figs. 6–7, Supplementary Tables 1 and 2. Figure 3d, h show the theoretical prediction and experimental statistics of the misalignment angle of hBN and graphene grown on different substrates. The results indicated that the predicted misalignment angles of all the islands are strikingly consistent with the measured values within the range of measurement error (±0.5 degree). This excellent agreement validates our theoretical model used to determine the alignment of 2D material islands on arbitrary surfaces.

**Fig. 4:**
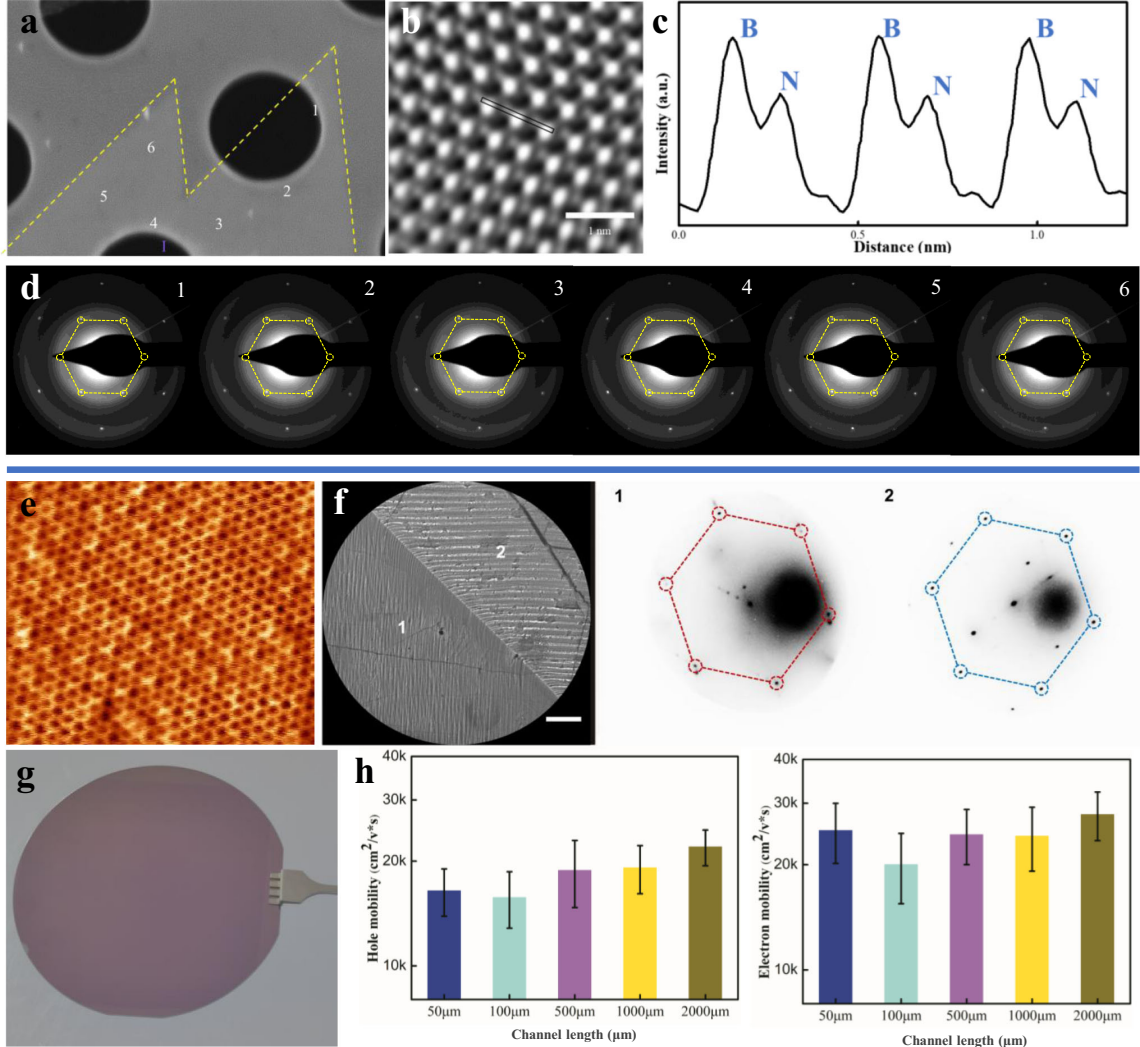
Characterization of single-crystal hBN and graphene films. **a** SEM image of two aligned hBN domains merging together; samples were then transferred onto a TEM copper mesh. **b** The corresponding STEM-ADF image of the merging area around zone I. **c** The intensity profile measured at the marked position in I. **d** SAED measurements of the different positions around the merging area. **e** STM image of the grown single-crystal graphene film. **f**, LEEM and selected-area μLEED measurements of the single-crystal graphene grown on a twinned Cu foil (scale bar 3 μm). **g** Single crystal graphene film transferred to 4-inch SiO_2_/Si wafer. **h** Carrier mobility of holes and electrons from the single-crystal graphene-based FET test with different channel sizes (channel width 10 μm, length 50–2000 μm. The error bar is the standard deviation of raw data).

The aligned domains on twinned Cu substrates eventually coalesce to form a thin film. To confirm this, we performed high-resolution TEM to characterize the merging area of domains at the atomic scale. The scanning TEM-annular dark-field (STEM-ADF) image and intensity profile of the merging area in Fig. 4a–c show the homogeneous nature of the single layer hBN film, which is also verified by atomic force microscopy (AFM) measurement in Supplementary Fig. 8a, b. Hexagonal selected area electron diffraction (SAED) patterns were collected at different positions around the merging area of two well-aligned hBN domains, shown in Fig. 4d. The Second Harmonic generation (SHG) mapping clearly show that the parallel hBN domains are seamlessly stitched together, as shown in Supplementary Fig. 9a. Two sets of SAED patterns were observed in the merging area of two misaligned hBN domains in Supplementary Fig. 9. In addition, hydrogen etching was used to analyze the crystallinity of the samples on the macroscopic scale^42,43^. No etching lines were observed between aligned hBN domains in Supplementary Fig. 8e. In contrast, for domains formed by the merging misaligned hBN islands, etched boundaries are clearly visible, as shown in Supplementary Fig. 8f. The above results confirm that well-aligned hBN domains can be seamlessly stitched into a large single-crystal hBN film.

For graphene, the D band Raman map of two merged islands in Supplementary Fig. 10b can be used to further confirm the absence of defects at the merging area. Low-energy electron microscopy (LEEM) and micro-low energy electron diffraction (μLEED) measurements were also performed. The μLEED patterns of selected areas confirmed that the graphene grains are precisely aligned on both sides of the twin boundary in Fig. 4f. Besides, the recently proposed seamless coalescence criteria^44^ were adopted and we further confirmed the seamless coalescence of well-aligned graphene islands, nucleated on both sides of the Cu twin boundary in Supplementary Fig. 11a. Aligned graphene domains were seamlessly stitched together to form a single crystal film (the graphene growth process, Supplementary Fig. 12), as shown in Fig. 4g. The crystallinity of randomly chosen 5 × 5 mm^2^ areas of the as-grown graphene/Cu sample was tested by low-energy electron diffraction (LEED) (Supplementary Figs. 11d, e). The Raman spectrum of the continuous as-grown graphene film showed a high I_2D_/I_G_ ratio and a negligible D peak (Supplementary Fig. 11c). Furthermore, a series of field effect transistor (FET) devices with 10 μm-wide channels and channel lengths ranging from 50 μm to 2 mm were fabricated to evaluate the electrical transport characteristics^8,45^, as shown in Supplementary Fig. 13a, b. No large changes in the carrier mobility of the devices (Supplementary Fig. 13c, d) were observed when the graphene channel length was varied by two orders of magnitude (Fig. 4h). The average electron and hole mobility was found to be 2.2 × 10^4^ cm^2^V^−1^s^−1^ and 2.8 × 10^4^ cm^2^V^−1^s^−1^ at room temperature, while the corresponding values for FETs with polycrystalline graphene is only 0.6 × 10^4^ cm^2^V^−1^s^−1^ and 0.8 × 10^4^ cm^2^V^−1^s^−1^.

It is worth to noting that, in our proposed method, the twin structures are highly stable after annealing and generally don’t change during graphene/hBN growth. The in-situ SEM observation of the graphene growth process shown in Supplementary Fig. 14 clearly confirms this. In addition, our experimental observations show that, different from the well explored normal grain boundaries^46,47^, no apparent grooves appear near a twin boundary of the Cu foil and no enhanced graphene nucleation near the twin boundary area are observed (Supplementary Fig. 15). This can be understood by the high stability of the twin boundaries and the closed packed atomic arrangement near the twin boundaries.

## Discussion

In summary, we established a geometrical model to determine the alignment of 2D material islands on an arbitrary substrate and validated it through numerous experimental observations. Based on our concise model, we predicted that single-crystal 2D materials can be epitaxially grown on various twinned substrates. Experimentally, we have successfully synthesized a number of differently twinned Cu foils and demonstrated the growth of single-crystal hBN and graphene films on these substrates. In addition to demonstrating our theoretical model, this study provides a viable approach to synthesize 2D films with tailored grain boundaries for future applications.

## Methods

### Modeling techniques

Calculations of the interfacial formation energy of graphene with different edges attached to Cu steps were performed within the framework of the DFT-D3 method^48,49^ as implemented in the Vienna ab initio simulation package (VASP)^50,51^. Exchange-correlation functions were treated according to the generalized gradient approximation (GGA)^52^, and the interaction between valence electrons and ion cores was modeled by the projected augmented wave (PAW) methods^53^. Along the direction of the interface of a graphene edge attached to a Cu step edge, the lattice mismatch between the graphene edge and the step edge in the supercell was limited within 3.0%, and the graphene lattice was used to avoid extra strain on graphene. Along the direction perpendicular to the step edge, the distance between parallel step edges was maintained at a constant value of ~20 Å. In the out of plane direction, the vacuum space between neighboring images was kept at least 12 Å to avoid periodic imaging interactions. A force on each atom of less than 0.01 eV/Å with an energy convergence of 10^−4^ eV, was used as the criterion for structural relaxation. Computational details are discussed in the supplementary information.

### Preparation of twinned Cu substrate

In this study, we used commercial oxygen-free high conductivity (OFHC) Cu foils with a purity of 99.99 wt% from CHINALCO Shanghai Copper Company. The cold-rolled oxygen-free high purity polycrystalline Cu foils had a thickness of 46 µm and hardness of 132 HV (measured using a load of 50 g). X-ray diffraction measurements showed that the initial texture is composed of typical rolling components of S {123}<634> (34.6%, volume fraction), Cu {112}<111> (16.6%), Brass {011}<211> (19.7%) and Goss {011}<001> (5.8%). The annealing treatments were conducted in a 100 mm-diameter furnace in H_2_/Ar (30/500 sccm) ambient gas under atmospheric pressure. A quartz tube with a diameter of 80 mm was used to load the samples into the furnace. The Cu foil was bent to fit the quartz tube and annealed at 1010 °C for 1 h; a central hardness indent was created using a pyramid-shaped diamond hardness indenter under 300 g load and 10 s dwell time at the center of the Cu foil.

### hBN epitaxy

hBN film was grown on a twinned Cu substrate by a chemical vapor deposition method. BNH_4_ (Alfa Aesar, 99.9%) served as the B and N source. The growth was performed after completing the Cu annealing process to obtain a twinned Cu substrate. The gas ambient was H_2_/Ar (10/200 sccm) under 200 Pa pressure for 45 min at 1010 °C to grow a single crystal hBN film, while heating the precursor at 65 °C within 10 min using a water bath.

### Graphene growth

Graphene film was grown on twinned Cu substrate by a chemical vapor deposition. Growth was performed after completing the annealing process to obtain twinned Cu. The gas ambient was CH_4_/H_2_/Ar (0.1/40/500 sccm) under atmospheric pressure and growth was carried out for 15 min at 1010 °C to obtain single crystal graphene film. Using a CH_4_ concentration of 185 ppm, it took approximately 15 min to fully cover the Cu surface with monolayer single-crystal graphene. To investigate the grain structure of graphene, the reaction was terminated before the Cu surface was completely covered by adjusting the experiment time from 1 to 15 min.

### Raman spectroscopy measurements

Raman spectroscopy and mapping were performed using a He/Cd laser (inVia Reflex, 532 nm wavelength) excitation source at 1 mW power. The objective lens 50X was used and the spatial resolution was 1 μm. The accumulation time for each spectrum was 0.5 s for image scanning and 1 s for a single spectrum.

### Etching of graphene and hBN

Etched hexagonal holes appeared on graphene after 10-min etching at 800 °C with 10/500 sccm H_2_/Ar under atmospheric pressure. To visualize the etched areas, each sample was oxidized in air on a hot plate at 190 °C for 5 min. Oxygen passed through the etched regions to react with the underlying Cu substrate to form CuO while the Cu substrate under other areas remained unreacted.

### SEM and EBSD measurements

SEM and EBSD measurements were performed with a JEOL 7640/Oxford EBSD nano system. The working voltage was 10 kV.

### TEM measurements

TEM was performed on twinned Cu structures and merging hBN domains. The twinned Cu was cleaned by electrochemical polishing, followed by conventional ion milling using a Gatan Model 691PIPS. HR-TEM images and the corresponding SAED patterns were obtained on an aberration-corrected TEM (FEI Titan3 G2) with an acceleration voltage of 300 kV. A double-tilt analytical holder was used. In the twin structure measurement, rotation angles α and β were 20.56° and −7.75°, respectively. After α and β rotations, the twin boundary was perpendicular to the sample holder, making the twin structure easier to observe.

### LEED and LEEM/μLEED measurements

Low Energy Electron Diffraction (LEED) images were obtained from single-crystal graphene film on twinned Cu foil using a BDL600IR system from Omicron. A 1 cm × 1 cm section of the sample was cut and attached to a metal holder and introduced to a chamber under ultrahigh vacuum with operating pressure at 1 × 10^−10^ Torr. The LEEM/μLEED measurement was performed on The LEEM III system (Technishe Universitat, Clausthal) with operating pressure at 4 × 10^−10^ Torr.

### Transfer of hBN and graphene films

After CVD growth, hBN and graphene were transferred onto a 300-nm SiO_2_/Si substrate by a wet transfer process using 4 wt% polymethyl methacrylate (PMMA) in ethyl lactate. The PMMA was spin-coated onto the graphene at 500 rpm for 5 s and 4000 rpm for 40 s and the samples were then heated on a hot-plate at 90 °C for 10 min. The samples were then treated in an oxygen plasma etching chamber (30 W, 15 sccm) for 60 s to remove the hBN and graphene from the backside of the Cu foil. The treated samples were allowed to float on the surface of a Cu etching solution (10 mL H_2_O_2_, 15 mL HCl and 300 mL DI water) for 4 h. The PMMA/hBN(Graphene)/Cu was transferred into another solution (250 mL DI water, 10 mL HCl), allowed to stand for 30 min. and soaked in DI water for 30 min. The obtained PMMA/ hBN (Graphene) was then transferred onto the target substrate. The PMMA/ hBN(Graphene)/substrate was heated on a hot plate at 100 °C for 30 min, followed by soaking in acetone, alcohol, and finally in DI water.

### Graphene FETs fabrication

Graphene field-effect transistors were fabricated by ultraviolet light lithography. Graphene films on Cu were transferred onto a 300 nm SiO_2_/Si substrate by wet transfer method. Electron beam evaporation and thermal evaporation methods were used to form electrical contacts (Cr/Au, 3 nm/45 nm), while oxygen plasma etching (30 W, 15 sccm, 3 ~ 5 min) was used to produce graphene ribbons. A series of FETs of different sizes (width 10 μm, length 50 μm, 100 μm, 500 μm, 1000 μm, 2000 μm) were produced for our research.

### Carrier mobility measurements

The transfer curve (*I*_*ds*_*-V*_*g*_) of the graphene FET was measured on a probe station with a Keithley 4200 semiconductor characterization system at room temperature under normal pressure. The widely-used Kim model based on nonlinear fitting was used to calculate the mobility:

1 $${R}_{{total}}=\frac{{V}_{{ds}}}{{I}_{{ds}}}$$

2 $${R}_{{to}{tal}}={R}_{{contact}}+\frac{L}{W\sqrt{{n}_{0}^{2}+n{[{V}_{g}]}^{2}}\mu e}$$

3 $${V}_{g}-{V}_{{Dirac}}=\frac{e}{{C}_{{ox}}}n+\frac{{{\hslash }}{v}_{F}\sqrt{\pi n}}{e}$$

where *I*_*ds*_ is the current between the source and drain electrodes, *V*_*ds*_ is the drain voltage with source grounded, *V*_*g*_ is the back-gate voltage, *R*_*total*_ is the resistance between the source and drain electrodes, *R*_*contact*_ is the contact resistance of the source and drain, *L* is the graphene channel length ranging from 50 μm to 2 mm, *W* is the graphene channel width fixed at 10 μm, *n*_*o*_ is the carrier density at the Dirac point (residue carrier density), *n* is the carrier density induced by the back-gate bias away from the Dirac point, *μ* is the carrier mobility, *e* is the electron charge, *V*_*Dirac*_ is the gate voltage at the Dirac point, *C* is the dielectric capacitance of silicon oxide, *ħ* is Planck constant, and *v*_F_ is the Fermi velocity of carriers in graphene, which is 1.0 × 10^8^ cm/s. Three parameters, namely, the mobility (*μ*), residue carrier density (*n*_*o*_), and contact resistance (*R*_*contact*)_. were fitted. To improve fitting consistency, we measured more than seven graphene FET devices for each channel length.

### In situ observation experiments

In situ CVD growth experiments were performed inside the chamber of a modified commercial ESEM (FEI Quantum 200). After sample loading, the chamber was pumped out to around 10^−3^ Pa, purged with pure nitrogen and pumped again to 10^−3^ Pa successively for several times. Under CVD growth conditions, the pressure is six orders of magnitude higher than the base pressure and constitutes mostly H_2_ (99.9995% purity) and C_2_H_4_ (99.95% purity). The substrate is pretreated 50 μm-thick Cu foil (99.999% purity). CVD growth was performed at temperatures ranging from 700 degree to 1000 degree, with a pressure in the chamber ranging from 10^−3^ Pa to 25 Pa.

## Supplementary information

Supplementary Information

## Data Availability

The data that support the findings of this study are available from the corresponding authors upon reasonable request.
