# Supplementary material for: Single-crystal two-dimensional material epitaxy on tailored non-single-crystal substrates
Source: Nat Commun. 2022 Apr 1;13:1773. doi: 10.1038/s41467-022-29451-w (PMC8975884; doi:10.1038/s41467-022-29451-w)
Supplement: Supplementary file 1 — Supplementary Information [file 41467_2022_29451_MOESM1_ESM.pdf]

Title: Single-Crystal Two-Dimensional Material Epitaxy on Tailored Non-Single-Crystal Substrates

Authors: Xin Li# [1,2], Guilin Wu# [3,4], Leining Zhang# [5,6], Deping Huang[1], Yunqing Li[1,2], Ruiqi Zhang[1,2], Meng Li[7], Lin Zhu[7], Jing Guo[3], Tianlin Huang[3], Jun Shen[1], Xingzhan Wei[1], Ka Man Yu[8], Jichen Dong[5], Michael S. Altman[8], Rodney S. Ruoff [5,6,9,10], Yinwu Duan[11], Jie Yu[11], Zhujun Wang[12], Xiaoxu Huang* [3,13], Feng Ding* [5,6], Haofei Shi* [1,2], Wenxin Tang[7]

Affiliations:

[1] Chongqing Key Laboratory of Multi-Scale Manufacturing Technology, Chongqing Institute of Green and Intelligent Technology, Chinese Academy of Sciences, Chongqing, 400714, P.R. China

[2] University of Chinese Academy of Sciences, No.19(A) Yuquan Road, Shijingshan District, Beijing, 100049, P.R. China

[3] International Joint Laboratory for Light Alloys (MOE), College of Materials Science and Engineering, Chongqing University, Chongqing, 400044, P.R. China

[4] Beijing Advanced Innovation Center for Materials Genome Engineering, University of Science and Technology Beijing, Beijing, 100083, P.R. China

[5] Center for Multidimensional Carbon Materials (CMCM), Institute for Basic Science (IBS), Ulsan 44919, Republic of Korea

[6] Department of Materials Science and Engineering, Ulsan National Institute of Science and Technology (UNIST), Ulsan 44919, Republic of Korea

[7] Electron Microscope Center, Chongqing University, Chongqing, 400044, PR China

[8] Department of Physics, The Hong Kong University of Science and Technology (HKUST), Clear Water Bay, Kowloon, Hong Kong, PR China.

[9] School of Materials Science and Engineering, Ulsan National Institute of Science and Technology (UNIST), Ulsan 44919, Republic of Korea

[10] School of Energy and Chemical Engineering, Ulsan National Institute of Science and Technology (UNIST), Ulsan 44919, Republic of Korea

[11] Chongqing Key Laboratory of Graphene Film Manufacturing, Chongqing, 401329, P.R. China

[12] Shanghai Tech University, 93 Middle Huaxia Road, Pudong, Shanghai, 201210, P.R. China

[13] Shenyang National Laboratory for Materials Science, Chongqing University, Chongqing 400044, P.R. China

# These authors were equal major contributors to this work

*Corresponding author. Email: xiaoxuhuang@cqu.edu.cn (X.H.); f.ding@unist.ac.kr (F.D.); shi@cigit.ac.cn (H.S.)

Contents

I. Supplementary Notes

1. The definition of a coordinate system and the three Euler angles ($\varphi1$, Φ, $\varphi2$) of crystal rotation

2. Definition of θ and ψ of a twin in the Cu foil

3. Interfacial formation energies of various graphene edges attached to different types of Cu step edges

4. Determining the direction of the longest (dominant) <110> step edge segment on an arbitrary Cu surface

5. Calculating the misalignment angle between graphene islands grown on both sides of a twin boundary

II. Supplementary Figures and Tables

Supplementary Figure 1 Atomic structure of a <110>/60º twin boundary of an FCC crystal.

Supplementary Figure 2 Surface indices of <1 1 1>/60º twins with different orientations.

Supplementary Figure 3 Definition of $\Delta\theta$ and $\Delta\psi$.

Supplementary Figure 4 Alignment analysis of graphene on an arbitrary Cu surface.

Supplementary Figure 5 Tailored twinned Cu foil with (1 1 6)/(1 1 1) orientation.

Supplementary Figure 6 Alignment of hBN islands on various twinned Cu surfaces.

Supplementary Figure 7 Alignment of graphene islands on various twinned Cu surfaces.

Supplementary Figure 8 Characterization of obtained hBN.

Supplementary Figure 9 Coalescence between aligned or misaligned hBN domains.

Supplementary Figure 10 The characterization of merging graphene islands.

Supplementary Figure 11 Characterization of the seamless coalescence of aligned graphene islands and following single-crystal graphene film.

Supplementary Figure 12 Time evolution of graphene islands growth on the (1 1 6)/(1 1 1) twinned Cu surface.

Supplementary Figure 13 Graphene carrier mobility characterizations using FETs devices.

Supplementary Figure 14 The in-situ SEM observation of graphene growth on twinned copper substrate.

Supplementary Figure 15 Characterization of graphene grown near a twin boundary.

Supplementary Table 1 Experimentally obtained (Δ_{Exp}) and theoretically predicted (Δ_{Map}) misalignment angles of graphene islands on various twinned Cu foils.

Supplementary Table 2 Experimentally obtained (Δ_{Exp}) and theoretically predicted (Δ_{Map}) misalignment angles of hBN islands on various twinned Cu foils.

I. Supplementary Notes

1. The definition of a coordinate system and the three Euler angles (φ1, Φ, φ2) of crystal rotation

φ_1, Φ, φ_2 are the three Euler angles used to describe the orientation of a rigid body with respect to a reference coordinate system. Here we use the Cu foil as the reference coordinate system, in which ND is the normal direction, TD is the transverse direction and RD is the rolling direction of the Cu foil. The coordinate system of a Cu grain in the Cu foil is defined with its three orthogonal axes along the [100], [010] and [001] directions of the FCC Cu lattice.

The three Euler angles that describe the orientation of a Cu grain in the Cu foil are defined by three rotational operations: the rotation about the Z axis by φ_1 degrees, rotation about the new X axis by Φ degrees, and rotation about the updated Z axis by φ_2, at the end of which the two coordinate systems coincide.

The surface index of a Cu grain in the Cu foil can be calculated by using the three Euler angles measured by electron back-scattered diffraction (EBSD). Any rotation can be implemented by multiplying a rotation matrix; the details are shown in the following table. Given a unit vector **U** = (x, y, z), where $x^2 + y^2 + z^2 = 1$, the rotation matrix G of a rotation around the vector **U** by angle θ is:

$$G=\begin{bmatrix} \cos\theta + x^2(1-\cos\theta) & xy(1-\cos\theta) - z\sin\theta & xz(1-\cos\theta) + y\sin\theta \\ xy(1-\cos\theta) + z\sin\theta & \cos\theta + y^2(1-\cos\theta) & yz(1-\cos\theta) - x\sin\theta \\ xz(1-\cos\theta) - y\sin\theta & yz(1-\cos\theta) + x\sin\theta & \cos\theta + z^2(1-\cos\theta) \end{bmatrix} \quad (1)$$

In order to calculate the surface index of a Cu grain in the Cu foil, we chose three arbitrary points of the Cu grain on the surface of foil, whose coordinates in the coordinate system of the single-crystal Cu grain are A (X_1, Y_1, Z_1), B (X_2, Y_2, Z_2), and C (X_3, Y_3, Z_3). The normal direction <*hkl*> can be obtained by $\overrightarrow{AB} \times \overrightarrow{BC}$.

$$h = \frac{(Y_2 - Y_1)(Z_3 - Z_2)}{(Z_2 - Z_1)(Y_3 - Y_2)} \quad (2)$$

$$k = \frac{(Z_2 - Z_1)(X_3 - X_2)}{(X_2 - X_1)(Z_3 - Z_2)} \quad (3)$$

$$l = \frac{(X_2 - X_1)(Y_3 - Y_2)}{(Y_2 - Y_1)(X_3 - X_2)} \quad (4)$$

Therefore, the surface index of a Cu grain is determined based on the three Euler angles.

2. Definition of θ and ψ of a twin in the Cu foil

A twin in a Cu foil has three degrees of freedom. The rotation along the axis normal to the surface of the foil is trivial and the other two degrees of freedom are described by θ and ψ here. θ denotes the angle between the Cu foil surface and the twin boundary plane, and ψ denotes the rotation angle of the twin crystal around the <1 1 1> co-axis of the two single crystals. In Supplementary Figure 2, we show the <1 1 1>/60º twin boundary with θ = 90º and ψ = 30º as an example. Because of the 6-fold symmetry of the <1 1 1> twin boundary, the range of ψ is limited from -30 to 30º.

The orientations of the grains on both sides of a grain boundary can be confirmed by the experimental measured Euler angles, and then the quality of the twin crystal can be evaluated, as depicted in Supplementary Figure 3. In Supplementary Table 1 and 2, we see that Δθ values for all the samples are less than 1º and $\Delta\psi$ are all around 60º, indicating that all the observed grain boundaries are <111>/60º twin boundaries.

3. Interfacial formation energies of various graphene edges attached to different types of Cu step edges

The orientation of a graphene island grown on a Cu substrate is determined by its interaction with the substrate, which includes the weak van der Waals interaction between the graphene bulk and substrate, and the strong graphene edge-metal surface interaction. During nucleation or when the graphene island is very small, graphene edge-metal surface interaction is the dominant interaction because of the strong chemical bonding between the edge carbon atoms and the metal surface, and the large edge to area ratio of the graphene island. On a metal surface with step edges, such as high index surfaces, graphene tends to nucleate near a step edge and hence, the graphene edge-metal step-edge interaction critically determines the alignment of graphene island on the metal surface. Here, we define the interfacial formation energy of a graphene edge attached to a Cu step edge as:

$E_{edge/Cu} = (E_{edge} - E_{bonding})/L$,

where E_{edge} is the formation energy of a freestanding graphene edge and $E_{bonding}$ is the bonding energy of a graphene edge attached to a step edge of the Cu substrate, defined by:

$E_{bonding} = E_{Cu} + E_{gra} - E_T$,

Here, E_T is the total energy of the system (graphene on the Cu substrate), and E_{Cu} and E_{gra} are the energies of the freestanding Cu substrate and graphene, respectively.

In this work, we compared the interfacial formation energies of various graphene edges attached to different Cu step edges. Using the graphene zigzag edge (0° in the map) and Cu <110> (0° in the map), respectively, as reference directions to indicate the directions of graphene edges and Cu step edges, each configuration can be uniquely described by the angle of the graphene edge and that of the metal step edge.

The density functional theory (DFT) calculated interfacial formation energy map is shown in Supplementary Fig. 4b. The results clearly show that all interfacial formation energy minima are located along the diagonal line of the map. This indicates that a graphene zigzag edge attaching to the longest (dominant) <110> step edge segment of the Cu surface is the most stable configuration of graphene on an arbitrary Cu surface.

4. Determining the direction of the longest (dominant) <110> step edge segment on an arbitrary Cu surface

The longest <110> step edge segment is the direction that determines the zigzag edge orientation of the graphene islands. In order to calculate the direction of the longest (dominant) <110> step edge segment of an arbitrary Cu surface, we chose a single-crystal Cu foil with an arbitrary orientation that can be measured by the three Euler angles. By comparing the angles between the six <110> axes of the face-centered cubic (FCC) Cu and the Cu foil surface, the <110> axis with the smallest angle and its projected direction on the surface, or the direction of the longest <110> step edge segment of an arbitrary Cu surface, can be obtained.

The projected direction of a <110> axis of a Cu grain (denoted by $\overrightarrow{BA}$) on the Cu foil can be obtained by calculating the projections of two points of the axis, represented by A (a_1, b_1, c_1) and B (a_2, b_2, c_2), on the Cu foil surface.

The plane equation of the Cu foil surface in the single-crystal coordinate system is defined as:

$$hx + ky + lz + d = 0. \quad (5)$$

Here, $d = -(h \cdot X_4 + k \cdot Y_4 + l \cdot Z_4)$, where (X_4, Y_4, Z_4) are the coordinates of a point on the Cu foil surface.

The coordinates of the projection of an arbitrary point (a, b, c), (P_a, P_b, P_c), can be calculated as

$$P_a = a + t \cdot h \tag{6}$$

$$P_b = b + t \cdot k \tag{7}$$

$$P_c = c + t \cdot l \tag{8}$$

where $t = -\frac{h \cdot a + k \cdot b + l \cdot c + d}{h^2 + k^2 + l^2}$.

Then the projected direction $\overrightarrow{B'A'}$ can be calculated as:

$$\overrightarrow{B'A'} = A'(P_{a1}P_{b1}P_{c1}) - B'(P_{a2}P_{b2}P_{c2}) \tag{9}$$

Therefore, the included angle α between a <110> axis and the Cu foil surface can be calculated as:

$$\cos\alpha = \frac{\overrightarrow{BA} \cdot \overrightarrow{B'A'}}{|\overrightarrow{BA}||\overrightarrow{B'A'}|} \tag{10}$$

5. Calculating the misalignment angle between graphene islands grown on both sides of a twin boundary

Since a graphene zigzag edge prefers to attach to the longest <110> step edge segment of the Cu surface and therefore, the misalignment angle between two graphene islands grown on both sides of a twin boundary is the same as the angle between the two longest <110> step edge segments on both sides of the twin boundary. Here, the theoretical misalignment angles between graphene islands grown on both sides of the twin boundary, Δ_{Map}, are calculated. Comparing with experimentally measured values, Δ_{Exp}, we conclude that our theoretical predictions are in very good agreement with experimental measurements, as listed in Supplementary Table 1 and 2.

II. Supplementary Figures and Tables

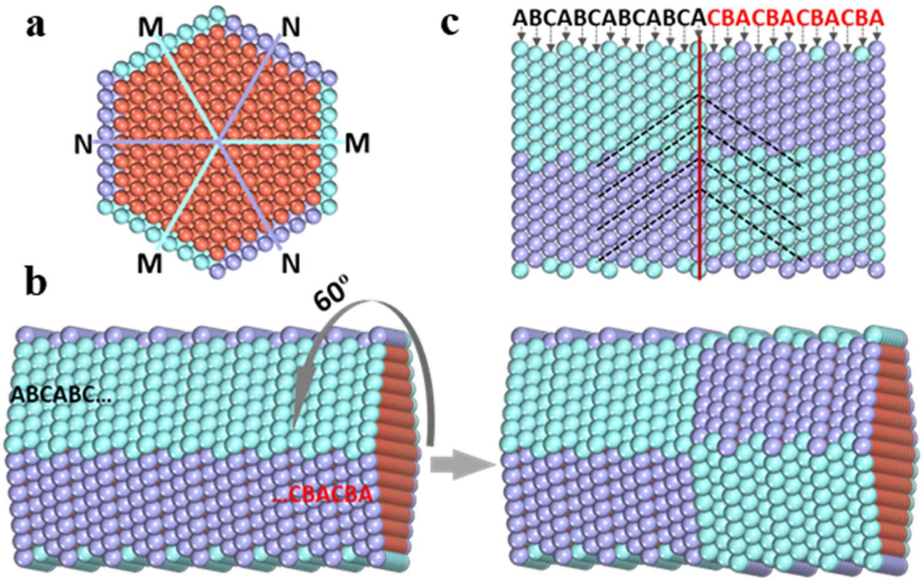

Supplementary Figure 1 Atomic structure of a <110>/60º twin boundary of an FCC crystal. a, A hexagonal prisms along a 3-fold symmetric <111> direction of a FCC crystal, resulting in two types of side facets. **b,** A <110>60º twin boundary in an FCC crystal can be generated by rotating half of the prism perpendicular to a <111> axis by 60 degree. **c,** Side view of the twinned prism, where the stacking order of atoms of …ABC|A|CBA… crossing the twin can be clearly seen. Besides the change of stacking sequence, there is no disorder introduced by the twin boundary. The <110>60º twin boundary are highly stable and has extremely formation energy in an FCC crystal. Generally, the <110>60º twin boundary are hardly to be annealed during the heat treatment of materials.

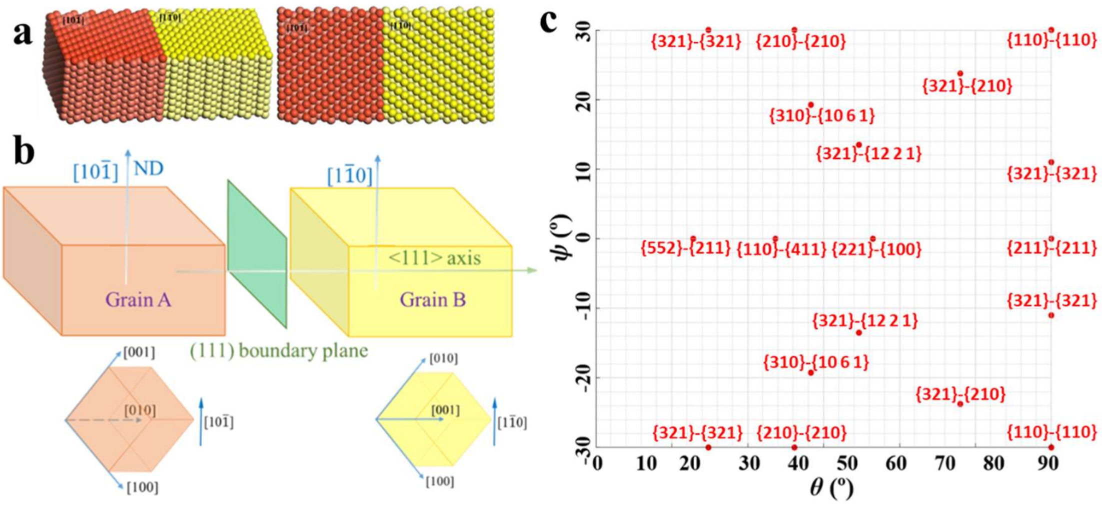

Supplementary Figure 2 Surface indices of <1 1 1>/60° twins with different orientations. a, Different views of the atomic structure of a Cu foil with θ = 90° and ψ = 30°. **b,** The orientations of the two single grains on both sides of the Cu foil of a. **c,** The map of the <111>/60° twin as a function of θ and ψ with some typical surface indices labelled.

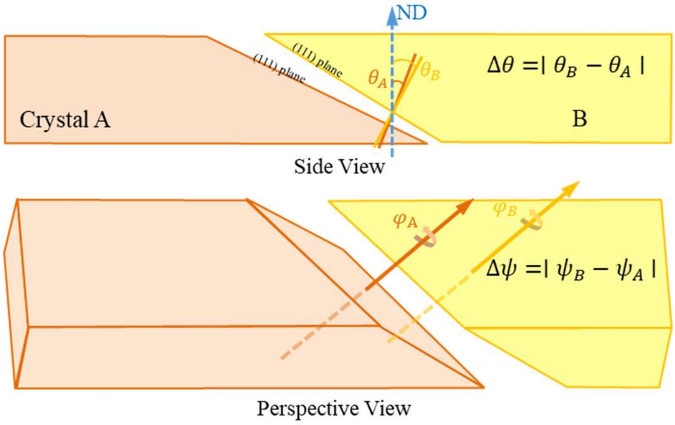

Supplementary Figure 3 Definition of $\Delta\theta$ and Δψ. θ_A and θ_B denote the angles of the (111) boundary plane of the crystal A (left) and B (right) to the Cu foil surface and ψ_A and ψ_B denote the rotation angles of crystal A and B around their <111> coaxis. For a perfect <111>/60° twin, $\Delta\theta = 0°$ and $\Delta\psi$ =60°.

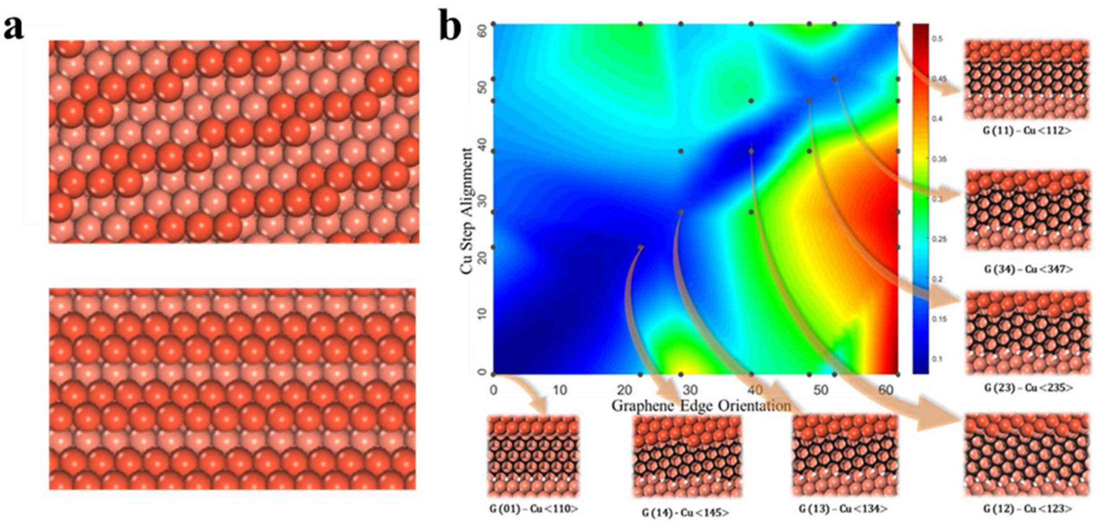

Supplementary Figure 4 Alignment analysis of graphene on an arbitrary Cu surface. a, Atomic configurations of two high-index surface of an FCC metal. **b,** Interfacial formation energy map of various graphene edges attached to different Cu steps. One graphene zigzag edge (0˚ in the map) and one Cu <110> (0˚ in the map) are used as references for the directions of graphene edges and Cu steps, respectively. Each configuration can be denoted by the angles of the graphene edge and the metal step relative to their respective reference directions (typical configurations are shown in the insets). The interfacial formation energy obtained from first principle calculations is denoted by colors shown in the color bar from 0 to 0.52 eV/Å.

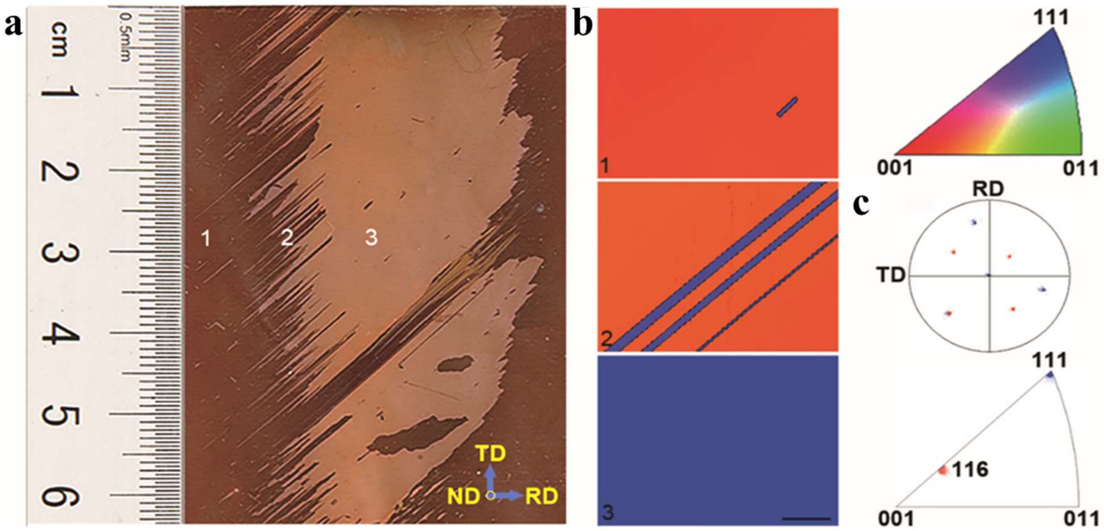

Supplementary Figure 5 Tailored twinned Cu foil with (1 1 6)/(1 1 1) orientation. a, Optical image of an oxidized twinned Cu foil. **b,** Representative EBSD maps of the Cu foil with a color legend. The scale bar represents 500 µm. **c,** Pole figure and inverse pole figure of the Cu foil.

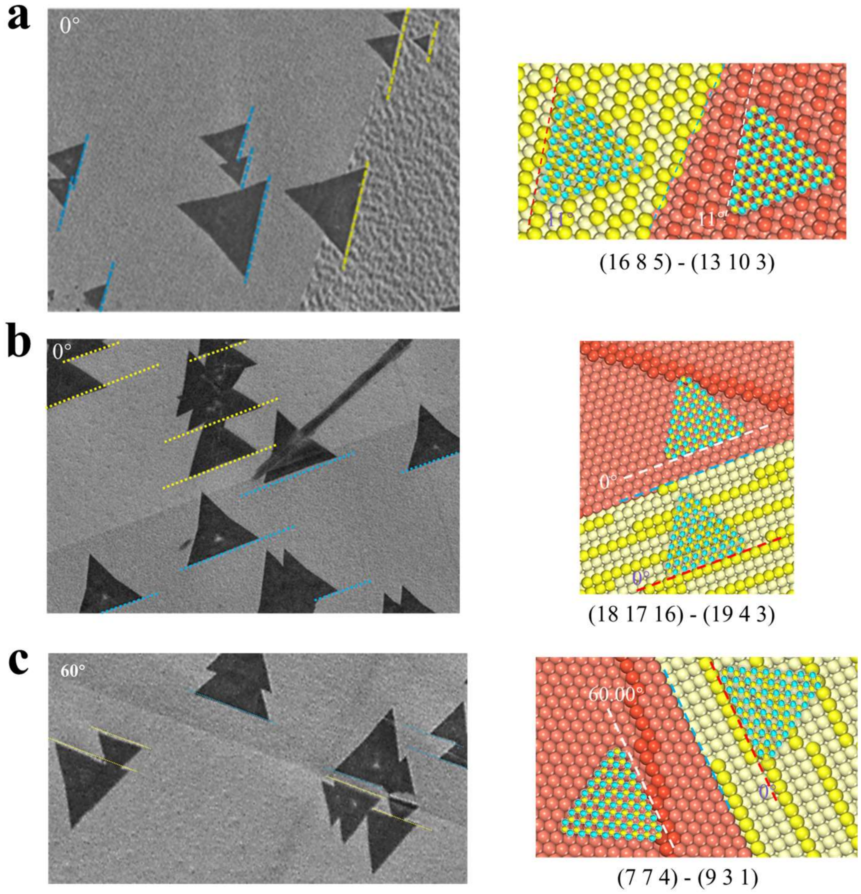

Supplementary Figure 6 Alignment of hBN islands on various twinned Cu surfaces. SEM images of typical experimental observed hBN islands alignment on twinned Cu surface (left panels) in comparison with the theoretical prediction (right panels). **a** and **b** present two parallel alignment on both sides of a twin boundary, while **c** presents a case of antiparallel hBN islands on both sides of a TB (corresponding to ⑦⑥③ in Supplementary Table 2). The surface indexes of the twin crystals were shown below each figure.

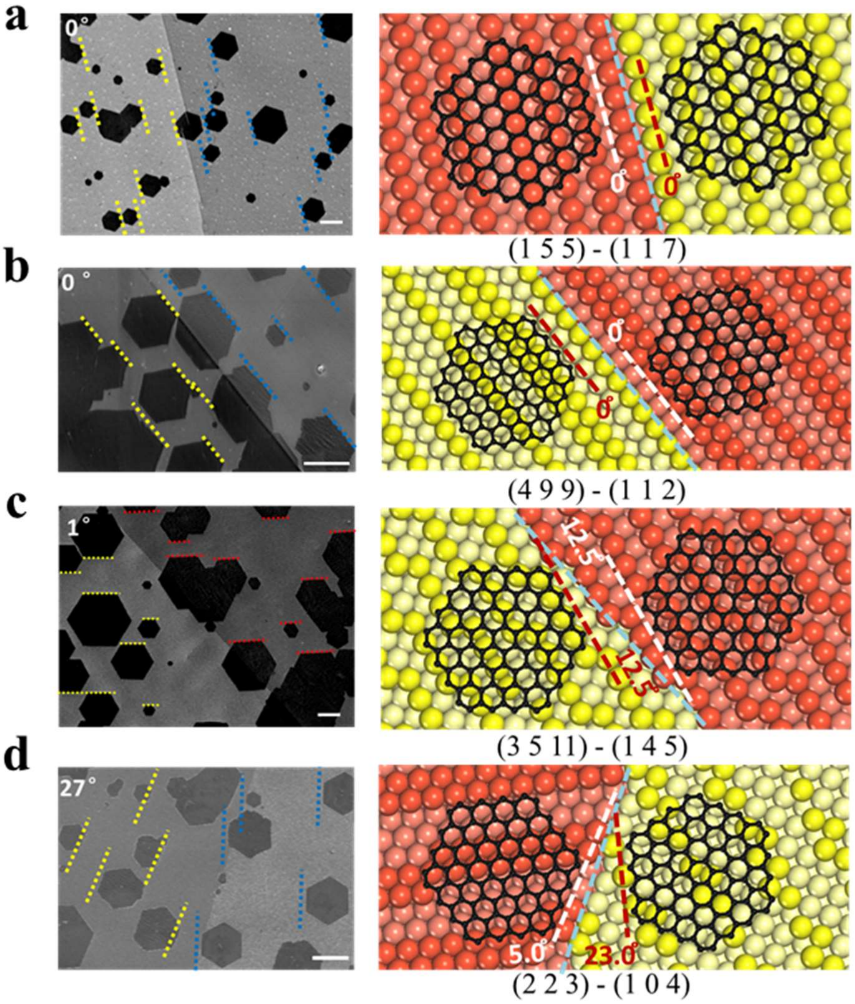

Supplementary Figure 7 Alignment of graphene islands on various twinned Cu surfaces. SEM images of typical experimental observed graphene islands alignment on twinned Cu surface (left panels) in comparison with the theoretical prediction (right panels). **a** and **b** are the alignment cases (corresponding to ⓬ and ⓮ in Supplementary Table 1), while **c** and **d** are typical misaligned cases (corresponding to ❼ and ❿ in Supplementary Table 1). The angle between the graphene zigzag edge and twin boundary is marked in the theoretical diagram. The surface indexes of the twin crystals were shown below each figure. The lengths of the scale bars are 10 μm.

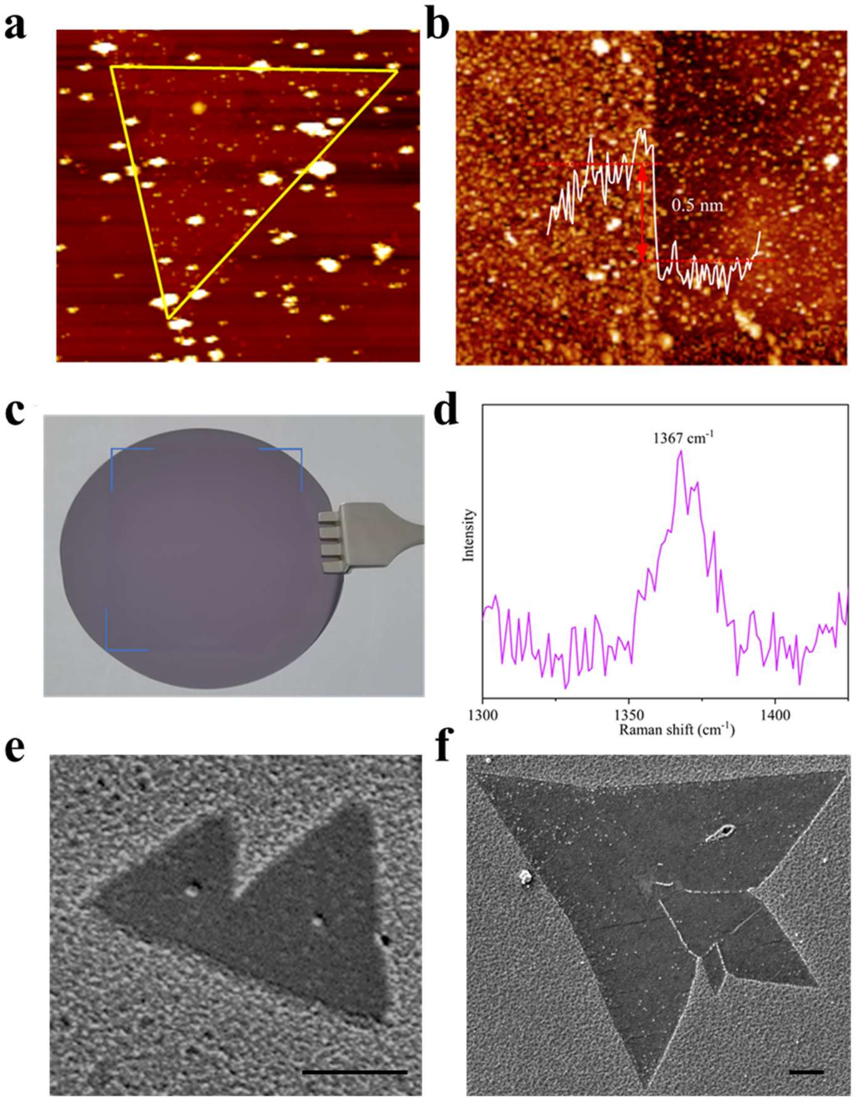

Supplementary Figure 8 Characterization of obtained hBN. (a-b) AFM measurement of obtained hBN island. **c,** Single crystal hBN film transferred to 2-inch SiO_2/Si wafer. **d,** Raman spectral of obtained hBN film. **e-f**, SEM images of aligned and misaligned hBN domains after hydrogen etching process. The scale bar is 5μm.

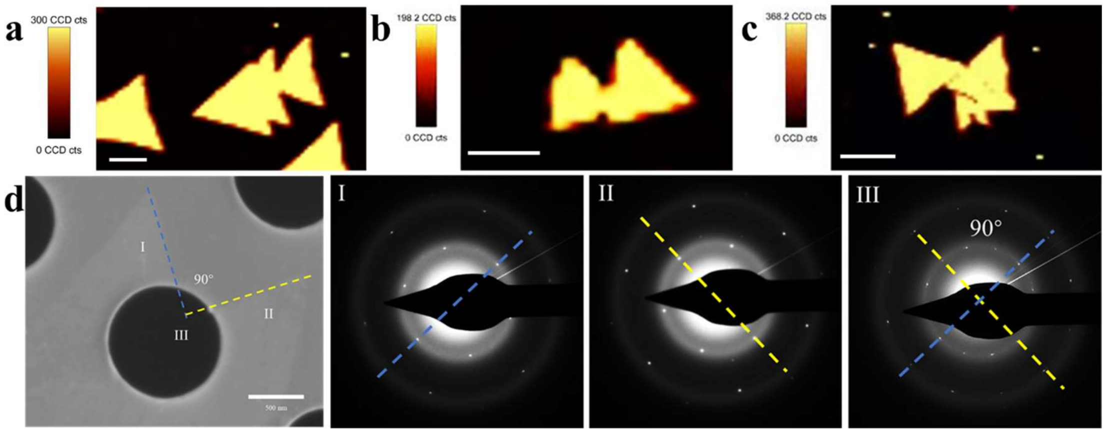

Supplementary Figure 9 Coalescence between aligned or misaligned hBN domains. The SHG mapping of (a-b) parallel and (c) unparallel hBN domains (Scale bars are 8μm). **d,** TEM and SAED measurement of two hBN domains with the misalignment angle of 90°. I, II and III are the corresponding SAED images. The results indicated that the misaligned hBN domains merged together show the diffraction patterns of two domains.

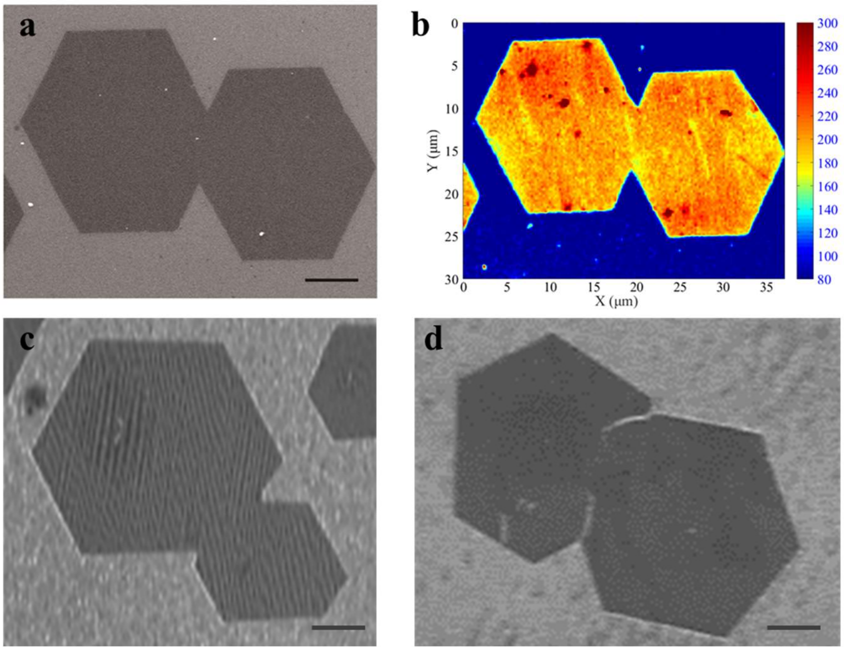

Supplementary Figure 10 The characterization of merging graphene islands. a, SEM image of two merged aligned graphene islands. **b,** D band Raman map of two merged aligned graphene islands. **c-d**, SEM images of two merged aligned and misaligned graphene islands after hydrogen etching, the scale bar is 25μm.

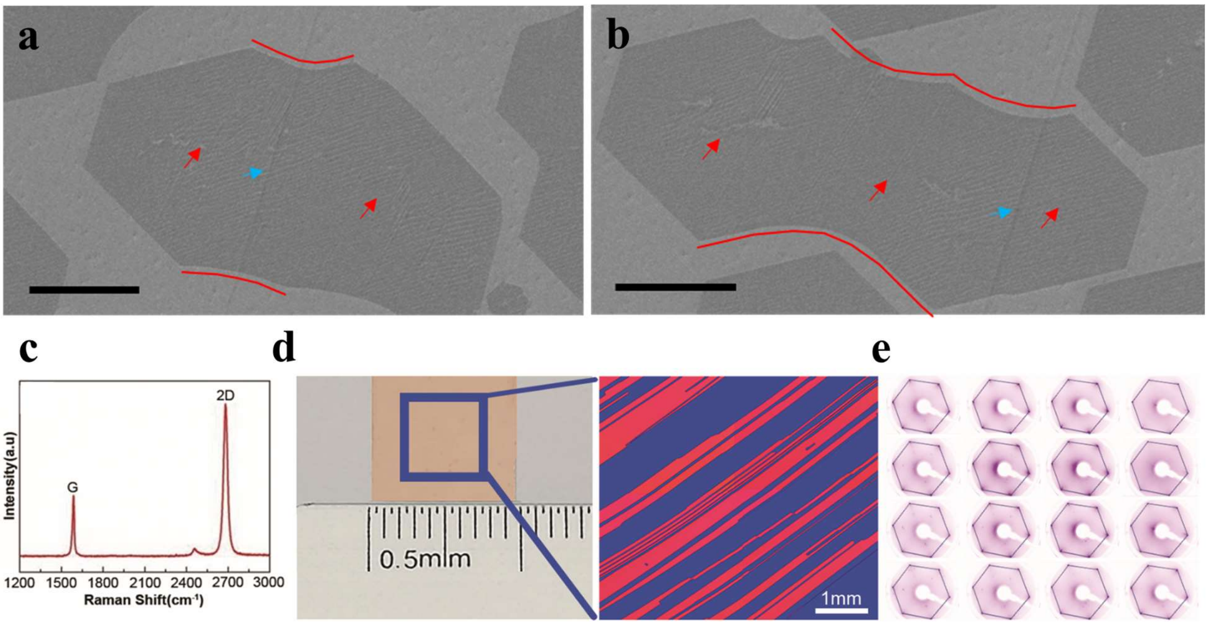

Supplementary Figure 11 Characterization of the seamless coalescence of aligned graphene islands and following single-crystal graphene film. a, The coalescence of two aligned graphene islands nucleated on both sides of the Cu twin boundary. **b,** The coalescence of three graphene islands, two of which were nucleated from the left side of the twin boundary and the third one from the right side. The blue arrow indicates the twin boundaries and the red arrows point to the nucleation regions of the graphene islands. Recently, it was proved that the observed smoothen concave corners of aligned graphene islands can be used as a criterion for the judgement of seamless coalescence. In our results, the smoothen concave corners formed by the coalescence of the graphene islands from both sides of the twin boundary clearly show a seamless coalescence. The scale bar is 10 μm. **c,** Raman spectrum of obtained graphene film. **d,** Optical image and EBSD map of a twinned Cu foil and LEED patterns of the graphene grown on it. **e,** Series LEED measurements of the single-crystal graphene grown on the twinned Cu foil.

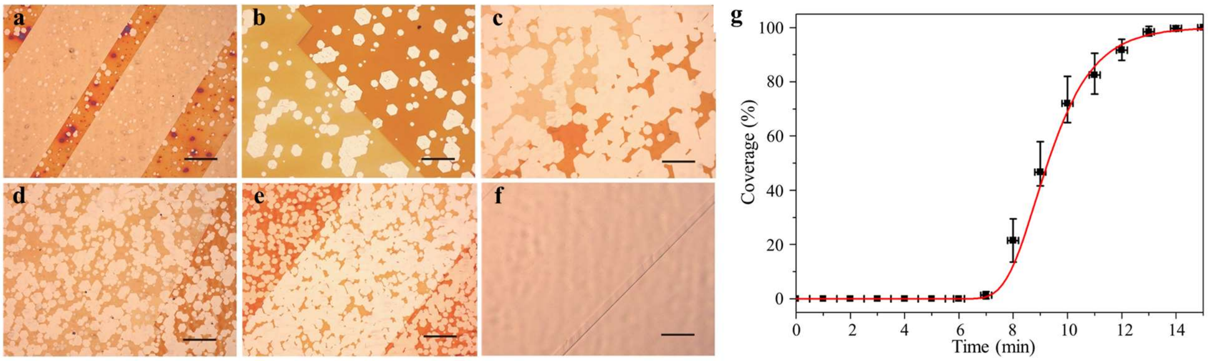

Supplementary Figure 12 Time evolution of graphene islands growth on the (1 1 6)/(1 1 1) twinned Cu surface. Optical images of graphene with different growth time: **a,** 7 min, **b,** 8 min, **c,** 9 min, **d,** 10 min, **e,** 12 min, and **f,** 15 min. As seen in the obtained optical images, the graphene nuclei were first nucleated on the (1 1 6)/(1 1 1) twinned Cu foil, followed by the growth of the hexagonal graphene islands which merged with each other to form a continues graphene film. We found that one edge of these hexagonal islands has the same orientation, indicating the growth of graphene islands with expected zigzag edges orientations is consistent with the DFT theoretical calculations. The scale bar is 50 μm. **g,** Growth process of graphene on the tailored twin Cu foil, where the error bars represent the standard deviation of the statistical means from multiple measurements (n is 3 or more).

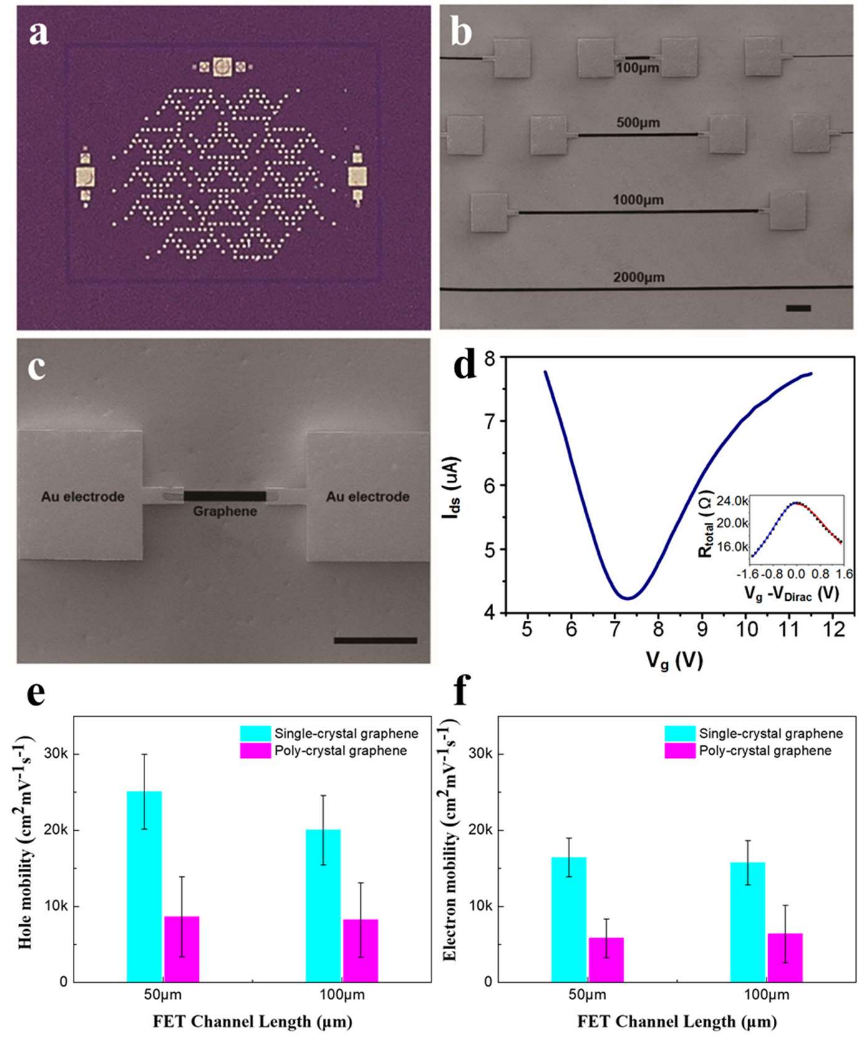

Supplementary Figure 13 Graphene carrier mobility characterizations using FETs devices. a, Optical image of graphene-based FETs array on SiO_2/Si substrate (white dots are electrodes). **b,** SEM images of graphene-based FETs of different sizes. The scale bar is 100 μm. **c,** SEM image of a 100 μm long graphene-based FET. The scale bar is 100μm. **d,** The electrical properties of a representative single-crystal graphene FET: the transfer characteristics (I_{ds} vs V_g). The inset is R_{total} vs V_g-V_{dirac}. **e-f**, The carrier mobility of single-crystal and polycrystalline graphene films after transferred to SiO_2/Si substrate using the same processes.

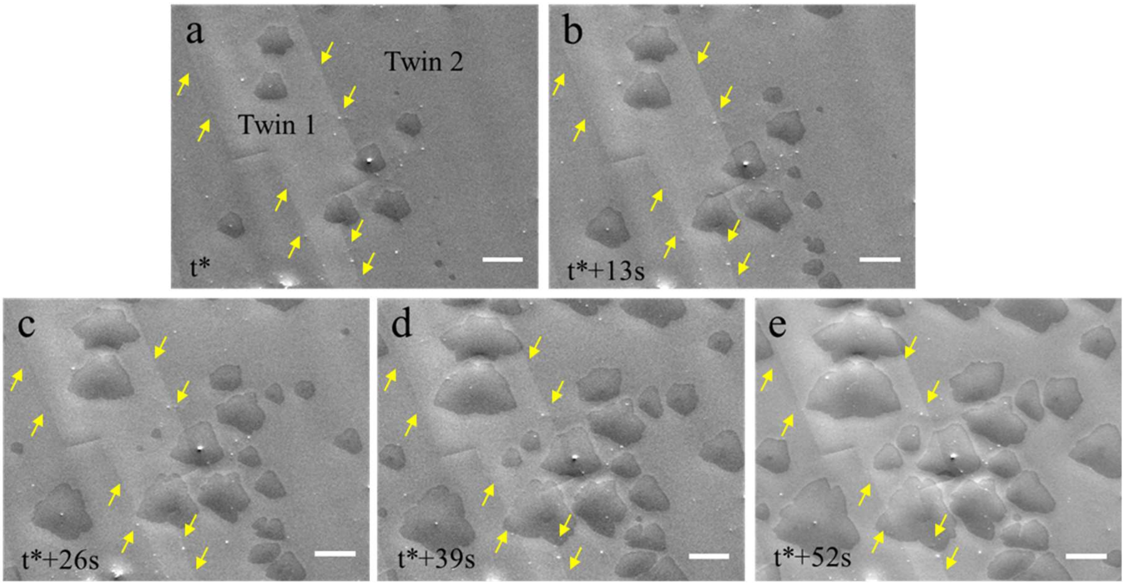

Supplementary Figure 14 The in-situ SEM observation of graphene growth on twinned copper substrate. a-e, SEM images of the nucleation and growth of graphene domains recorded at different times. Twin boundaries are pointed by arrows. It can be clearly seen that all the twin boundaries did not move during the whole period. (Scale bar is 10 μm)

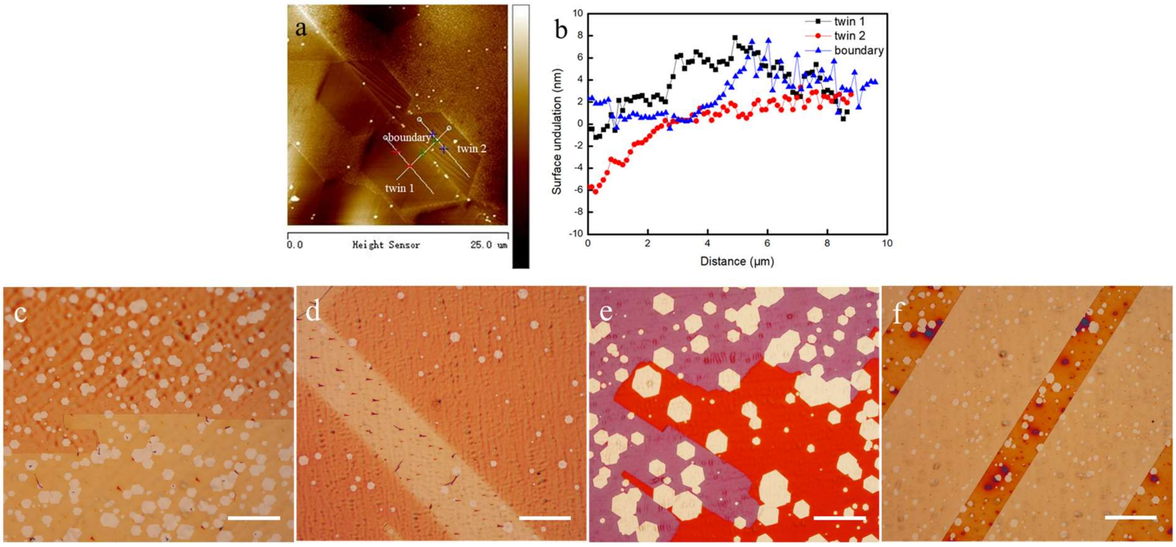

Supplementary Figure 15 Characterization of graphene grown near a twin boundary. a-b, AFM image of graphene domains grown on twinned Cu substrate and the surface undulation of different area around the twin boundary. There is no groves and significant height changes across the twin boundary. **c-f,** Optical images of graphene domains grown on Cu substrates with different twin densities (Scale bar is 100 μm). The nucleation density of graphene near twin boundaries is same as that on terrace.

Supplementary Table 1 Experimentally obtained (Δ_{Exp}) and theoretically predicted (Δ_{Map}) misalignment angles of graphene islands on various twinned Cu foils.

No.	Measured Euler Angles			Characterization of the twins				Graphene misalignment Analysis	
	φ_1 (°)	ϕ (°)	φ_2 (°)	θ (°)	ψ (°)	$\Delta\theta$ (°)	$\Delta\psi$ (°)	Δ_{Exp}(°)	Δ_{Map}(°)
1-A	221.00	12.20	44.70	66.94	-0.07	0.02	59.79	0±0.5	0.00
1-B	283.30	52.90	41.20	66.96	-59.86				
2-A	227.90	12.80	37.70	57.32	15.14	1.00	59.66	27.2 ± 0.5	27.21
2-B	15.50	37.00	37.50	58.32	-44.52				
3-A	136.10	39.10	19.20	47.77	-80.08	0.51	59.17	0±0.5	0.00
3-B	284.20	9.60	90.00	48.28	-20.91				
4-A	233.10	46.70	46.80	67.83	-81.76	0.36	59.71	0±0.5	0.08
4-B	144.40	14.50	75.70	67.47	-22.05				
5-A	139.10	41.20	14.50	80.71	5.11	0.73	60.37	11.0±0.5	11.00
5-B	222.00	35.30	88.10	81.44	-55.26				
6-A	141.10	39.30	12.50	80.28	2.82	0.13	59.72	11.0±0.5	11.28
6-B	224.90	35.50	84.80	80.41	-56.90				
7-A	262.1	28.4	58.8	27.75	-14.10	0.62	61.23	1±0.5	0.00
7-B	354.2	39.9	13.6	27.13	47.13				
8-A	64.5	36.6	53.7	67.05	-39.79	0.68	61.00	25±0.5	26.42
8-B	312.8	22.6	15.6	67.73	21.21				
9-A	123.1	41.4	46.2	63.62	47.57	0.01	60.19	27±0.5	27.47
9-B	236.3	14	81	63.63	-12.62				
10-A	71.3	39.8	80.8	82.08	-31.61	0.35	60.24	10±0.5	11.03
10-B	349.3	37.6	6	81.73	28.63				
11-A	126.9	31.4	27.4	95.01	-9.10	9.8	0.19	3±0.5	2.16
11-B	281	41	59.2	85.21	-9.29				
12-A	51.9	44.3	87.1	33.25	-58.66	0.61	60.13	0±0.5	0.00
12-B	142	20.9	42.7	33.86	1.47				
13-A	236.4	44.9	10.7	42.8	-59.12	0.15	59.86	0±0.5	0.00
13-B	336.3	12.1	42.6	42.65	0.74				
14-A	261.8	36.6	45.4	18.14	-0.77	0.1	59.02	0±0.5	0.00
14-B	335.5	47	23.9	18.04	58.25				

Note: i-A and i-B (i=1, 2, …, 14) represent the Cu surface on both sides of the twin boundary, (φ_1, Φ, φ_2) are Euler angles obtained from EBSD measurements; θ and ψ define the orientation of the twinned Cu surface.

Supplementary Table 2 Experimentally obtained (Δ_{Exp}) and theoretically predicted (Δ_{Map}) misalignment angles of hBN islands on various twinned Cu foils.

No.	Measured Euler Angles			Characterization of the twins				hBN misalignment Analysis	
	φ_1 (˚)	ϕ (˚)	φ_2 (˚)	θ (˚)	ψ (˚)	$\Delta\theta$ (˚)	$\Delta\psi$ (˚)	Δ_{Exp}(˚)	Δ_{Map}(˚)
1-A	315.6	50.5	36	62.24	59.46	1.21	56.73	60±0.5	60.00
1-B	271.4	6.7	24.1	61.03	2.73				
2-A	110.7	52.3	41.2	73.05	116.86	1.70	122.70	60±0.5	59.92
2-B	269.9	17.4	63.8	71.35	-5.84				
3-A	261	52.2	41.5	66.84	59.07	0.26	60.30	60±0.5	60.00
3-B	193.8	11.9	50.5	66.59	-1.23				
4-A	44.4	49.5	44.8	67.84	55.19	0.26	60.30	0±0.5	0.15
4-B	324.3	13.6	65.5	67.58	-5.11				
5-A	107.1	52.4	46.1	72.87	120.91	0.34	120.37	0±0.5	0.00
5-B	289.7	17.8	43.3	72.53	0.54				
6-A	44.8	52.2	43.5	68.24	58.27	0.04	59.45	0±0.5	0.14
6-B	339.2	13.5	49.7	68.20	-1.18				
7-A	302.1	30.5	31.9	25.70	-15.38	0.20	60.45	0 ± 0.5	0.00
7-B	212.5	39.7	73.5	25.50	45.06				
